# Adrenomyeloneuropathy manifesting as adrenal insufficiency and bilateral lower extremity spastic paraplegia: A case report and literature review

**DOI:** 10.1097/MD.0000000000036946

**Published:** 2024-01-12

**Authors:** Yan Chen, Daojing Li, Peng Xu, Aimei Zhang, Xu Chen, Yun Chen

**Affiliations:** a Department of Clinical Medicine, Jining Medical University, Jining, China; b Department of Neurology, Affiliated Hospital of Jining Medical University, Jining, China.

**Keywords:** ABCD1 gene, adrenal insufficiency, adrenomyeloneuropathy, spastic paraplegia, X-linked adrenoleukodystrophy

## Abstract

**Rationale:**

Adrenomyeloneuropathy (AMN) is a variant type of X-linked adrenoleukodystrophy, and it is a genetic metabolic disease with strong clinical heterogeneity so that it is easily misdiagnosed and underdiagnosed. Moreover, most patients with AMN have an insidious clinical onset and slow progression. Familiarity with the pathogenesis, clinical features, diagnosis, and treatment of AMN can help identify the disease at an early stage.

**Patient concerns:**

We present a case of 35-year-old male, who was admitted to our hospital due to “immobility of the lower limbs for 2 years and worsening for half a year,” accompanied by skin darkening and hyperpigmentation of lips, oral mucosa, and areola since puberty.

**Diagnosis:**

The level of very long-chain fatty acids was high and genetic testing depicted that exon 1 of the ABCD1 gene had a missense mutation of C.761c>T, which was diagnosed as AMN.

**Interventions:**

Baclofen was administered to improve muscle tension combined with glucocorticoid replacement therapy.

**Outcomes:**

The condition was relieved after half a year.

**Lessons:**

The clinical manifestations of AMN are diverse. When patients with adrenocortical dysfunction complicated with progressive spastic paraplegia of lower limbs are involved, AMN should be highly suspected, and the determination of very long-chain fatty acids and genetic testing should be performed as soon as possible to confirm the diagnosis because early treatment can help prevent or delay the progression of the disease.

## 1. Introduction

X-linked adrenoleukodystrophy (X-ALD) is a recessive genetic disease. The gene location is Xq28, and the most accurate incidence of the disease is approximately 1:15,000.^[1]^ Typical X-ALD has an early onset age, mainly in children and adolescents, and involves the cerebral hemisphere. Adrenomyeloneuropathy (AMN) is a variant type of X-ALD that usually occurs in adult males and mainly involves the spinal cord. The clinical manifestation is progressive spastic paralysis of both lower limbs.

## 2. Case report

A 35-year-old male presented to our hospital because of “weakness of both lower limbs for 2 years and with aggravation for half a year.” Two years before admission, the patient developed lower-extremities inactivity without obvious inducement, manifested as a shaking sensation during walking, which was progressively aggravated. In the past 6 months, the patient felt stiffness and weakness in both lower extremities, increased inability to walk and defecate, prone to falls, occasional difficulty in urination, no obvious decrease in sexual function, numbness and pain in the limbs, and muscle atrophy. No significant change in body weight was observed since the onset. Since junior high school, the patient presented hyperpigmentation all over the body and gradually developed pigmentation on the lips, oral mucosa, gums, and areola. There was no addiction to alcohol or tobacco and no history of exposure to toxic substances. His parents were in good health, not consanguineous, and his mother had 6 siblings, none of whom had similar diseases.

**Figure 1.**
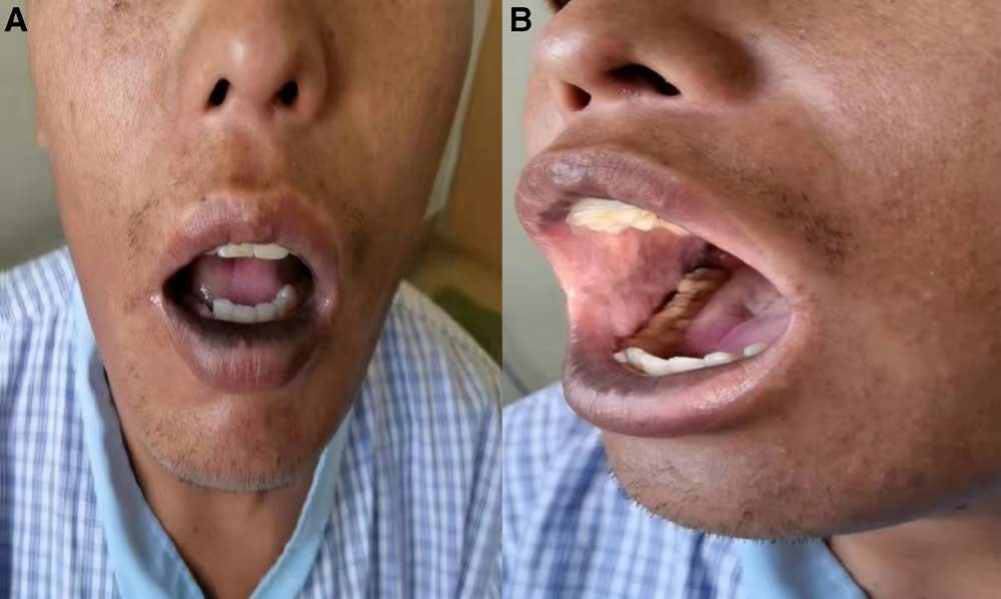
Pigmentation of the (A) lips and (B) mucous membranes in the mouth of the patient.

*Physical examination*: Normal consciousness; dark skin all over the body; pigmentation of the lips, intraoral mucosa, gums, and in the bilateral areola (Fig. 1); normal higher cortical functions; fluent speech; and cranial nerve examination (−). The muscle strength of both upper extremities was grade 5, while the muscle strength of both lower extremities was grade 4, the muscle tone of both upper extremities was normal, whereas the muscle tone of both lower extremities was high. The biceps reflex, triceps reflex, and radial reflex of both upper extremities were (++), the knee reflex was (++), the pain sensation was not decreased, the ataxic examination was normal, the kinesthesia and vibration sensation of both lower extremities were slightly decreased, the scissor gait, the double Bartholomew sign was positive, and Romberg sign was negative.

**Figure 2.**
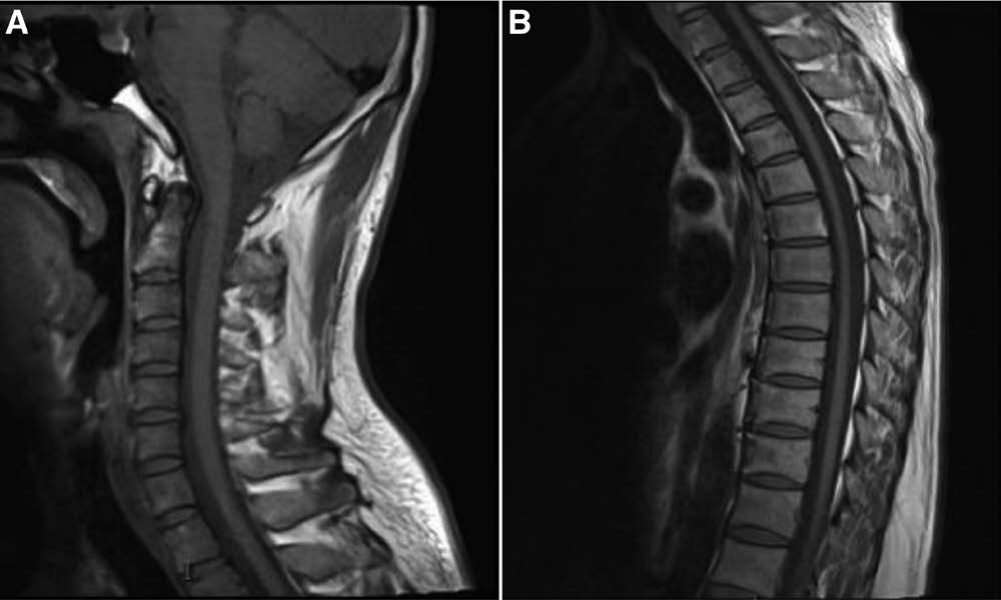
Spinal magnetic resonance imaging (MRI) of the patient without abnormal signal in the cervicothoracic medulla. (A) Mild posterior protrusion of the cervical 5 to 6 disc, (B) mild atrophy of the thoracic medulla.

*Ancillary tests*: Blood routine test, liver function, kidney function, cardiac enzymes, lipids, blood glucose, electrolytes, antinuclear antibody profile, tumor markers (CEA, CA153, CA199, AFP, and Cyfra21-1), ASO + RF + CRP, and urine and stool routine tests demonstrated no significant abnormalities. Hepatitis B, syphilis, and HIV tests were all negative. Magnetic resonance imaging (MRI) of the spinal cord suggested mild atrophy of the thoracic medulla and mild posterior protrusion of the cervical 5 to 6 disc (Fig. 2); MRI of the pituitary gland suggested normal size and shape; MRI of the cranial showed no significant abnormalities. Nerve conduction velocity demonstrated multiple peripheral nerve damage in both lower extremities, involving motor and sensory fibers, with myelin and axonal involvement. Thyroid function was normal. Plasma cortisol, serum adrenocorticotropic hormone and gonadotropic hormone levels were as follows: plasma cortisol (8 AM): 2.83 μg/dL (reference value: 4.2–38.4 μg/dL), serum adrenocorticotropic hormone: >440.400 pmol/L (reference value: 1.6–13.9 pmol/L), prolactin: 19.28 ng/mL (reference value 2.1–17.7 ng/mL), luteinizing hormone: 10.16 Miu/mL (reference value: 1.5–9.3 Miu/mL), follicle-stimulating hormone: 30.83 Miu/mL (reference value: 1.4–18.1 Miu/mL); progesterone, testosterone, estradiol, and 24-hour urinary free cortisol were within normal range.

*Other laboratory findings*: Complete serum very long-chain fatty acids (VLCFAs) and genetic testing indicated increased serum concentrations (c24:0-LPC): 0.764 μmol/L, reference range: 0.015–0.172 μmol/L; c26:0-LPC: 0.797 μmol/L, reference range: 0.008–0.085 μmol/L; C24:0 LPC/c20:0-LPC ratio of 3.322, reference range: 0.124–1.087; C26: 0-LPC/C20:0-LPC ratio of 3.465, reference range: 0.066–0.716; C24: 0-LPC/c22:0-LPC ratio of 7.14, reference range: 0.481–3.172.; C26: 0-LPC/C22: 0-LPC ratio of 7.449, reference range: 0.183–2.316. Molecular genetic test report: chromosomal location: chrX: 152991482; nucleotide change: c.761C>T; amino acid change: p. Thr254Met; exon/intron: Exon1; variant type: hemizygous; variant classification: missense mutation; American College of Medical Genetics and Genomics rating: pathogenic; father: no variant found; mother: no variant found. The patient was finally diagnosed with AMN and was treated with “low-dose glucocorticoids and baclofen.” At the 6-month follow-up, the limb weakness of the patient improved slightly, and the skin became significantly whiter and less pigmented than before.

## 3. Interpretation

This case concerns a young male patient with a chronic course of AMN. The main clinical manifestations of this case were spastic paraplegia with darkening of the skin and hyperpigmentation of the lips, intraoral mucosa, and areola starting during adolescence; decreased plasma cortisol levels and increased adrenocorticotropic hormone (ACTH) levels; subclinical peripheral nerve damage found in nerve conduction velocity; high serum VLCFAs detected; genetic testing showed a missense mutation in exon 1 of the ABCD1 gene with c.761C>T. The localization diagnosis was bilateral corticospinal tract + adrenal cortex + peripheral nerves. The qualitative diagnosis was first an inherited metabolic disease, AMN. This disease is extremely rare and easily misdiagnosed and underdiagnosed. Therefore, we reviewed the literature in the context of this patient to improve our understanding of this disease.

**Figure 3.**
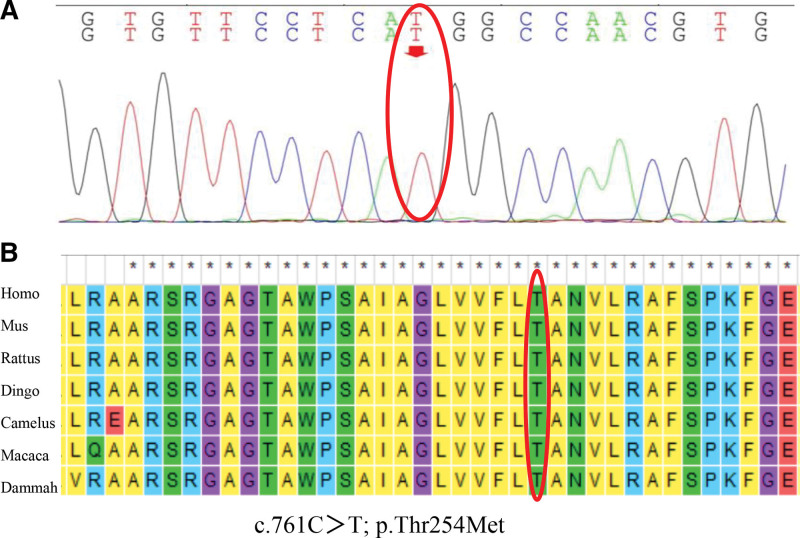
Mutation of c.761C>T in exon 1 in the *ABCD1* gene. (A) Missense mutation in the amino acid 254 from threonine to methionine. (B) The number 245 threonine acid is well conserved across species.

X-linked adrenoleukodystrophy (X-ALD) is a peroxisomal disease, which is also a disorder of lipid metabolism. The pathological alterations in ALD are caused by mutations in the ABCD1 gene (also known as the ALD gene), which is responsible for encoding the ABCD1 protein. This is a transporter protein located on the peroxisome membrane that specifically transports the very long-chain lipid coenzyme A synthase into the peroxisome to complete the oxidation of VLCFAs. When the ABCD1 gene is mutated, the structure and/or function of ABCD1 protein is altered, VLCFAs cannot enter the peroxisome to complete oxidative degradation, and the metabolism of LCFAs is impaired, leading to their toxic accumulation in organs and tissues and causing their dysfunction. AMN is a special variant of ALD, which is rare in the clinic. A summary of all the AMN cases reported worldwide between 1975 and 2019 found that the mutations most frequently occurred in exon 1 of the ABCD1 gene, and most of them were missense mutations.^[2]^ Genetic testing of our patient revealed a nucleotide mutation in exon 1 of the ABCD1 gene with a change from C to T in the coding region 761 (Fig. 3A), which resulted in a missense mutation in the amino acid 254 from threonine to methionine. The number 245 threonine acid is well conserved across species (Fig. 3B). The family of the patient was verified, and neither the father nor the mother had any mutation at this locus; thus, it was considered a de novo mutation. According to the American College of Medical Genetics and Genomics guidelines, the mutation type was rated as pathogenic. the carriage of this mutation in the normal population has not been reported to date.

AMN is a genetic metabolic disease with strong clinical heterogeneity. In a study of ALD/AMN patients in 20 pedigrees, no correlation was observed between the type of ALD mutation and clinical phenotype.^[3]^ Due to the inconsistent reactivity of different tissues and organs to the pathological accumulation of VLCFAs, patients at various stages of the disease show progressive organ damage rather than typical and complete syndromes. Most AMN cases reported in China are characterized by spastic paralysis of both lower limbs accompanied by peripheral nerve symptoms. Most patients with AMN exhibit intact cognitive function, with some presenting with mild cognitive dysfunction early on, presumably related to subtle myelin abnormalities that have not yet manifested as white matter lesions on the MRI of the brain. The early appearance of skin darkening and hyperpigmentation in this patient is consistent with adrenocortical insufficiency; the gradual appearance of spastic paraplegia of both lower extremities is consistent with spinal cord conduction tract damage; and the perfect EMG indicates subclinical peripheral nerve damage, which is consistent with the clinical features of AMN.

At present, the diagnosis of AMN is mainly based on the determination of VLCFAs content in serum, genetic detection, and even biopsy tissue testing. Because of its very strong clinical heterogeneity and diverse manifestations, the diagnosis of AMN cannot rely solely on clinical manifestations. MRI examination lacks the specificity of presentation. It can assist in the diagnosis when there are demyelinating changes in the brain white matter, suggesting a poor prognosis. Evoked potentials help to detect intracranial corticospinal tract conduction dysfunction in patients with pure AMN, even earlier than MRI changes. AMN, a non-inflammatory distal axonopathy that often involves the spinal cord and peripheral nerves, is often detected by nerve conduction studies that indicate axonal sensorimotor polyneuropathy. Recently, the rapid assessment of peripheral nerve morphology in patients with AMN has used high-resolution neurosonography, which shows significant multifocal regional nerve swelling with reduced echogenic intensity.^[4]^ Compared to electrophysiological examinations, high-resolution neurosonography has significant advantages due to its rapid and painless nature, which facilitates long-term follow-up and helps to reveal the evolution of peripheral neuropathy in patients with AMN.

Most patients with AMN have an insidious clinical onset and slow progression. When patients present with bilateral lower extremity spastic paraplegia as the main symptom, especially when there is no obvious abnormal signal in the white matter imaging of the brain, it is often misdiagnosed as hereditary spastic paraplegia. Hereditary spastic paraplegia is a group of genetic degenerative diseases of the nervous system that are inherited in multiple ways, with progressive bilateral lower limb spastic paraplegia and gait abnormalities as the main manifestations. Therefore, in patients with SP as the main clinical manifestation and without abnormal signals in the brain white matter, especially in male patients, further determination of VLCFAs, including plasma C26:0 and C24:0 concentrations and C26:0/C22:0 and C24:0/C22:0 ratios, is required. Additionally, a biopsy of the peroneal nerve to determine VLCFAs can be an important diagnostic basis. Elevated VLCFAs are important for the early detection and diagnosis of patients with asymptomatic AMN/ALD, but their elevated levels do not correlate with disease severity. Further, the levels of VLCFAs also do not distinguish typical X-ALD from AMN. The plasma C26:0 and C24:0 concentrations and C26:0/C22:0 and C24:0/C22:0 ratios were increased in our case compared to normal values, supporting the diagnosis of AMN/ALD.

It is worth mentioning that approximately 70% of patients with AMN will present with adrenocortical insufficiency and its corresponding symptoms, 15% of which will present with adrenocortical insufficiency before neurological symptoms manifest. This is because these patients may first consult the dermatology or endocrinology departments, and due to the lack of overall grasp of neurological diseases, it is easy to miss and neglect, resulting in a diagnosis delay. To secure an early diagnosis, clinicians must pay more attention to the comprehensive physical examination of each patient. On the other hand, specialists must have a complete and systematic understanding of the disease to avoid missing any suspicious clues, carefully exclude the corresponding disease, and pay more attention to finding the cause and targeting treatment in clinical work rather than staying on symptomatic treatment. The “darkening of the skin and hyperpigmentation of the lips, oral mucosa, and areola beginning in adolescence” attracted our attention. The diversity of human skin pigmentation is associated with high polymorphism of the melanocortin-1 receptor (MCIR), which is located on the surface of melanocytes. The alpha-melanocyte stimulating hormone and ACTH are agonists of this receptor and increase melanin synthesis. In contrast, the agouti-signaling protein blocks the binding of alpha-melanocyte stimulating hormone to melanocortin-1 receptor and exerts an antagonistic effect. We, therefore, hypothesized that persistently elevated serum adrenocorticotropic hormone levels may be an important driver of the hyperpigmentation of the skin of the patient, which was verified by the whitening of the skin and significant reduction in skin mucosal pigmentation after the ACTH supplementation.

There is no specific treatment for this disease. Good rehabilitation and assistive orthopedic devices can improve lower extremity flexion stiffness and help maintain gait and balance, thereby improving the quality of life of patients. In patients with adrenocortical insufficiency, early glucocorticoid replacement therapy can potentially improve skin-darkening symptoms. Dietary treatments that lower VLCFAs, such as Lorenzo oil, can reduce C26:0 levels without stopping the progression of neurological symptoms. In patients with mild neurological symptoms not associated with brain white matter damage, hematopoietic stem cell therapy can delay or avoid the disease progression. A treatment study with an infusion of autologous CD34 + cells transduced with Lenti-D lentiviral vectors suggested that Lenti-D gene therapy may be a safe and effective alternative to allogeneic stem cell transplantation^[5]^; however, further, follow-up is needed to fully assess the response duration and long-term safety. In short, in terms of treatment, when the neurological symptoms of the patient have not yet appeared or are mild, early treatment can prevent or delay the progression of the disease. Current treatment cannot improve existing neurological symptoms; therefore, patients with AMN need early diagnosis and treatment. Although the symptoms of hyperaldosteronism appeared early in our patient, early treatment was missed because of the failure to achieve a clear diagnosis with the plasma VLCFA level measurement and genetic testing. However, regular follow-up of patients with AMN is important to provide symptomatic treatment.

## 4. Conclusion

In summary, the clinical manifestations of AMN are diverse. When patients with adrenocortical insufficiency combined with progressive bilateral lower limb spastic paraplegia are encountered, AMN should be suspected, while the diagnosis of VLCFA determination and genetic testing should be improved as early as possible to achieve early diagnosis and treatment to prevent or delay the progression of this disease.

## Author contributions

**Investigation:** Peng Xu, Yun Chen.

**Resources:** Yan Chen.

**Supervision:** Daojing Li, Aimei Zhang.

**Writing – original draft:** Yan Chen, Xu Chen.

**Writing – review & editing:** Daojing Li, Yun Chen.
